# Adaptive laboratory evolution for increased temperature tolerance of the diatom *Nitzschia inconspicua*

**DOI:** 10.1002/mbo3.1343

**Published:** 2022-12-25

**Authors:** Alaina J. LaPanse, Tyson A. Burch, Jacob M. Tamburro, Jesse C. Traller, Agnieszka Pinowska, Matthew C. Posewitz

**Affiliations:** ^1^ Department of Chemistry Colorado School of Mines Golden Colorado USA; ^2^ Department of Quantitative Biosciences and Engineering Colorado School of Mines Golden Colorado USA; ^3^ Global Algae Innovations Lihue Hawaii USA

**Keywords:** adaptive laboratory evolution, diatom, microalgae, thermal tolerance

## Abstract

Outdoor microalgal cultivation for the production of valuable biofuels and bioproducts typically requires high insolation and strains with high thermal (>37°C) tolerance. While some strains are naturally thermotolerant, other strains of interest require improved performance at elevated temperatures to enhance industrial viability. In this study, adaptive laboratory evolution (ALE) was performed for over 300 days using consecutive 0.5°C temperature increases in a constant temperature incubator to attain greater thermal tolerance in the industrially relevant diatom *Nitzschia inconspicua* str. Hildebrandi. The adapted strain was able to grow at a constant temperature of 37.5°C; whereas this constant temperature was lethal to the parental control, which had an upper‐temperature boundary of 35.5°C before adaptive evolution. Several high‐temperature clonal isolates were obtained from the evolved population following ALE, and increased temperature tolerance was observed in the clonal, parent, and non‐clonal adapted cultures. This ALE method demonstrates the development of enhanced industrial algal strains without the production of genetically modified organisms (GMOs).

## Graphical abstract

In this study, adaptive laboratory evolution (ALE) was performed for over 300 days using consecutive 0.5°C temperature increases in a constant temperature incubator to attain greater thermal tolerance in the industrially relevant diatom *Nitzschia inconspicua* str. Hildebrandi. The adapted strain was able to grow at a constant temperature of 37.5°C; whereas this constant temperature was lethal to the parental control, which had an upper‐temperature boundary of 35.5°C before adaptive evolution. This ALE method demonstrates the development of enhanced industrial algal strains without the production of genetically modified organisms.

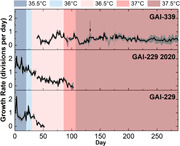

## 1 INTRODUCTION

Climate change modeling predicts that global surface temperatures will rise 5°C or more by the year 2100 (Sokolov et al., 2009). Currently, global temperatures have increased 1°C above preindustrial levels and the changing climate may lead to increasingly severe weather events, resource scarcity, and ocean level rise (Fawzy et al., 2020). Multiple renewable and carbon‐neutral technologies are needed to address global climate challenges and emission goals (Forsberg, 2009). One promising sustainable resource is photosynthetic algal biomass. Microalgae are compelling because of their short growth cycles, utilization of nonarable land, nonpotable water requirements, and biomass compositions (Demirbas, 2011; Foley et al., 2011). The proteins, carbohydrates, lipids, and pigments in algal biomass can be converted to economically valuable bioproducts, including fuels, biopolymers, plastics, nutraceuticals, food additives, animal feed, and antibiotics (Foley et al., 2011). The diatom *Nitzschia inconspicua* strain Hildebrandi is of interest for industrial cultivation due to its robust outdoor stability in large‐scale ponds, carbon use efficiency, and high lipid content (>50% under some conditions), which is a primary product for algal biofuels including sustainable aviation fuels (SAFs) (Levitan et al., 2014; Oliver et al., 2021).

Although promising, current algal productivities are frequently limited by environmental stressors in the “domesticated” conditions of industrial algae ponds, which include high O_2_ levels (super‐saturation) and high temperatures (≥37°C) (Weissman et al., 1988). To sustain high productivity under adverse conditions, strain improvements to address these physiological challenges are often necessary to improve stress tolerances, and product accumulation capabilities (Chisti, 2013). Direct genetic engineering and adaptive laboratory evolution (ALE) are both viable methods for algal strain improvements (Jeon et al., 2017; Zhang et al., 2019). Significant progress has been made in developing specific and reliable algal genetic engineering methods using gene insertion and/or CRISPR/Cas‐9, but timely phenotypic progress remains challenging in these systematic and targeted efforts (Dahlin & Guarnieri, 2016; Dahlin et al., 2019; Krishnan et al., 2020). While engineering is beneficial in understanding specific genes and gene knock‐outs (KOs), broader metabolic and systemic changes may be more effectively achieved through other, more global means, including ALE (Cheng et al., 2019; LaPanse et al., 2020; Li et al., 2015, 2018; Lohbeck et al., 2012, 2014; Sun et al., 2018a, 2018b; L. Wang et al., 2016; X. Wang et al., 2019).

ALE is well documented in yeast and bacteria as a means to gradually attain phenotypes of interest over time through the application of environmental selection pressure leading to novel genotypes with greater environmental fitness through the existence of random mutations within a population (Bennett et al., 1992, 1996; MoDeatherage et al., 2017; Monngold et al., 1996; Riehle et al., 2003). Recently, this technique has been applied to microalgae as a means of increasing tolerance to a variety of stresses (including temperature, salinity, herbicides, and flue gas components), increased utilization of substrates (including glucose, butanol, and dark conditions), increased valuable product yields (including lipids, biomass, and pigments), and increased growth rates (Cheng et al., 2019; LaPanse et al., 2020; D. Li et al., 2015; X. Li et al., 2018; SuLohbeck et al., 2012, 2014; Sun et al., 2018a, 2018b; L. Wang et al., 2016; X. Wang et al., 2019). The use and complexity of ALE experiments have evolved alongside directed genetic engineering methods like CRISPR/Cas‐9, and both methods have been shown to produce industrially beneficial phenotypes in algae (Barten et al., 2022; Krishnan et al., 2021). However, ALE is especially useful in selecting randomly occurring genotypes that may be difficult to isolate through single‐gene knock‐outs or insertions and can be advantageous for deployment because ALE variants are not typically regulated as genetically modified organisms (GMOs) (Zhang et al., 2021).

High‐temperature tolerance is a particularly relevant ALE objective for industrial algal strains. To achieve the maximal biomass productivity necessary for economic viability, cultivation in hot climates with year‐round warm temperatures and high sunlight is key. To accommodate these locations, algal strains must be minimally stressed and maintain high productivity levels in high‐temperature conditions. Initial efforts to improve algal temperature tolerance have been undertaken using both, acclimation over short periods (regulatory alterations) and adaptation over longer time frames (advantageous genetic alterations). While acclimation experiments may provide useful roadmaps for strain preconditioning before outdoor cultivation, adaptation will typically produce more stable phenotypes useful for long‐term outdoor production campaigns. Such phenotypes have been achieved in microalgae using ALE. A 2‐month ALE experiment in the cyanobacterium *Symbiodinium spp.* resulted in higher growth rates at 31°C compared to wild‐type (WT) controls, without low‐temperature growth trade‐offs (Chakravarti et al., 2017). Likewise, recent research in the green alga *Picochlorum spp.* increased the upper‐temperature boundary from 47.5°C to 49°C over 390 days of photobioreactor adaptive cultivation (Barten et al., 2022). These long‐term ALE experiments demonstrate the ability to achieve incremental, but important, gains in high‐temperature algal stress tolerance.

A subset of diatoms is of interest for industrial cultivation due to their high lipid content (used as a precursor to SAFs) and tolerance to high pH, and brackish media conditions, which can be useful when managing outdoor pests (Oliver et al., 2021). High‐temperature cultivation conditions are beneficial in achieving high productivity when growing diatoms outdoors, however, marine diatoms may not be naturally high‐temperature tolerant. Temperature adaptations have been achieved in diatoms through ALE. A year‐long ALE study in the marine diatom *Thalassiosira pseudonana* showed adapted growth rates at 32°C equivalent to the growth rate observed in the parent strain at the original temperature of 22°C (Schaum et al., 2018). ALE studies in diatoms showcase their ability to adapt to environmental conditions, much like green algae, bacteria, and yeast.

The industrially relevant diatom, *Nitzschia inconspicua*, has a maximal growth rate at ~25°C with temperatures above 36°C proving fatal under dilute culturing conditions. Successful outdoor algal cultivation sites will likely be located in warm climates with consistent daily photosynthetic radiation and minimal cloud cover (Sun et al., 2020). For *N. inconspicua* to be successfully cultivated outdoors in such conditions, greater thermal tolerance is necessary. Due to the complexity of thermal adaptation from a metabolic perspective, as well as regulatory limitations on the deployment of genetically modified organisms (GMOs) using recombinant DNA approaches, ALE was selected for strain thermal tolerance improvements (Varela Villarreal et al., 2020). In this study, we report a constant‐temperature tolerance increase of 2°C in *N. inconspicua* following nearly 1 year of continuous cultivation and ALE.

## 2 MATERIALS AND METHODS

### 2.1 Microorganism and culturing conditions

Strains of *N. inconspicua* were obtained from Global Algae Innovations (GAI) (Oliver et al., 2021). *N. inconspicua* strains GAI‐229 and GAI‐339 originated from separate clonal isolates of the parent organism after distinct outdoor cultivation campaigns. Initial constant temperature conditioning of GAI‐229 was undertaken in 2019 and 2020, and the resulting strains were designated GAI‐229 and GAI‐229 2020. GAI‐339 is derived from a separate clonal isolate of *N. inconspicua* obtained following a successful summer outdoor cultivation season.

Constant temperature cultures were maintained in a Multitron incubator with 200 µmol photons m^−2^ s^−1^ of photosynthetically active radiation (PAR) (16‐h light:8‐h dark cycle), 120 rpm shaking, and 1% CO_2_ supplementation. Cultures were maintained in 250 ml Erlenmeyer flasks with foam caps to allow air diffusion, and were diluted once per day, or once every few days, using brackish media as previously described (Chagoya et al., 2014; Jiang et al., 2015). Starting cultures were purified and streaked, and dilutions were performed in a sterile hood; however, cultures were not axenic at the start or throughout the experiment due to surface‐associated (phycosphere) bacterial organisms. Culture temperatures during incubator adaptation were measured using IR thermometer measurements and verified using alcohol thermometers placed alongside flasks in the Multitron incubator.

### 2.2 Adaptive laboratory evolution experimental conditions

To achieve a high‐temperature tolerant strain of *N. inconspicua*, multiple flasks were maintained using serial dilutions at steadily increasing temperatures for, in the longest case, nearly 300 days. Temperature adaptation began at the most stressful temperature set‐point possible without complete culture death and cell bleaching. Given the previously determined maximal temperature tolerance of the WT (parent) strain being 36°C, the internal temperature of the flasks began at 35.5°C. To prevent culture death during ALE, a ratchet methodology using gradual stepwise temperature increases of 0.5°C was undertaken. A temperature increase was triggered when the culture showed growth rate stabilization, with culture growth evaluated using optical density (OD_750_) measurements as a proxy for productivity (Reboud et al., 2007). Each ratcheting step was maintained for at least 10 days, with some steps being maintained for 30 or more days to allow for culture adaptation.

For ALE experiments, three strains of *N. inconspicua* were placed in the constant temperature incubator in triplicate to provide reproducibility metrics and to provide multiple starting points from which beneficial phenotypes could emerge. These strains, designated GAI‐229, GAI‐229 2020, and GAI‐339, were obtained from GAI as separate clonal isolates from successful outdoor cultivation campaigns at Global Algae's Kauai Algae Farm located in Lihue, Hawaii. GAI‐229 and GAI‐229 2020 both originated from the same GAI‐229 clonal isolate, but were maintained separately under long‐term laboratory cultivation and may have diverged genotypically as a result.

### 2.3 Cryopreservation and revival

Microalgal biomass was cryopreserved at −80°C as previously described (Elliott et al., 2012). Briefly, strains were grown in flasks to dense culture (above an OD_750_ of 1). A volume of 1.9 ml of dense culture was combined with 0.1 ml of the cryoprotectant dimethyl sulfoxide (DMSO) in 2 ml vials (5% DMSO %v/v). Vials were briefly mixed and then placed into a “Mr. Frosty” Nalgene Freezing Container containing isopropanol and maintained previously at 4°C. The “Mr. Frosty” containing samples for cryopreservation was placed into a −80°C freezer to achieve a temperature ramp of −1°C/min. Once samples inside the “Mr. Frosty” had been at −80°C overnight, they were transferred to a standard sample storage box and maintained at −80°C until revival.

Cryo‐revival of microalgal biomass was performed as previously described (Elliott et al., 2012). Frozen biomass vials were placed into a 37°C water bath to thaw for up to 5 min. Vials were then centrifuged at 1000 rpm for 5 min at 25°C. The supernatant with the DMSO cryoprotectant was aspirated and the pellet was washed once with a fresh medium and then centrifuged again. The cell pellet was then resuspended in 14 ml of dilute culture brackish medium and maintained in low light (<25 µE) conditions for 24 h. Following low light acclimation, the flask was grown in 100 µE light conditions on a shaker table at 110 rpm and diluted as necessary in dense culture brackish medium.

### 2.4 Clonal isolation

Single clones were isolated from *N. inconspicua* on agar plates using 1 µm filters [Cytiva (Formerly GE Healthcare Life Sciences) Whatman™ 47 mm Nuclepore™ Polycarbonate Track‐Etched Membranes]. Cultures were diluted to a concentration of 40–100 cells in a 10 ml sterile isotonic bicarbonate solution. Filters were first rinsed using 10 ml of sterile isotonic bicarbonate solution, then 10 ml of the dilute culture was filtered, and finally, the filter with culture was rinsed again using sterile bicarbonate. Filters were placed directly over agar plates and incubated in 100 µmol photons m^−2^ s^−1^ PAR, 1% CO_2_. Colonies were visible after a week to 10 days, restreaked on agar plates, then transferred to liquid media for phenotypic evaluation.

### 2.5 Microscopy

Microscopy was performed using a Nikon Eclipse E400 microscope using the CFI Plan Achromat Series objectives for phase contrast. Images were obtained using a high‐definition Nikon DS‐Fi1 camera head and processed using the Nikon Elements Br software (Nikon), as previously described (Elliott et al., 2012).

## 3 RESULTS AND DISCUSSION

### 3.1 Constant temperature adaptation increased the high‐temperature tolerance of *Nitzchia inconspicua*

Adaptive laboratory evolution (ALE) is a well‐described method for the selection of improved microbial and microalgal phenotypes over time (Bennett et al., 1996; Dragosits & Mattanovich, 2013; LaPanse et al., 2020; Sandberg et al., 2019). ALE is often characterized by an increase in the growth rate of a culture over time, as more environmentally competitive random mutations come to dominate a population. Stressful environmental conditions will select for subpopulations with greater stress tolerance, and in cases where a stress condition is lethal, a gradual, ratcheting increase in stress is effective in developing adaptive phenotypes (Bennett et al., 1992; Dragosits & Mattanovich, 2013).

**Figure 1:**
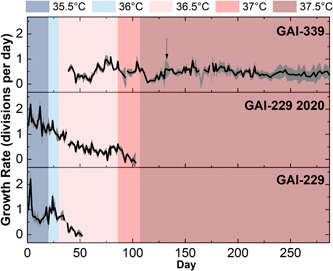
Growth rates (calculated from OD_750_ measurements) of multiple strains of *Nitzschia inconspicua* during constant temperature incubator‐based adaptation, with gray shading representing ±1 standard deviation between three biological replicates, arrow designating shift in biological replicates, and blue and red shading representing constant incubator temperature during adaptive laboratory evolution.

**Figure A1:**
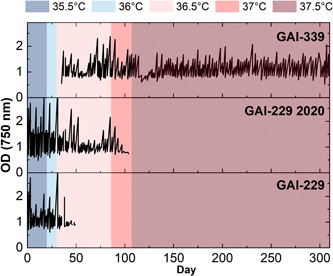
Average OD_750_ (between three biological replicates) measurements of multiple strains of *Nitzschia inconspicua* maintained in flasks through serial dilutions during constant temperature incubator‐based adaptation, with blue and red shading representing constant incubator temperature during adaptive laboratory evolution.

ALE began at 35.5°C with GAI‐229 and GAI‐229 2020, both of which immediately showed growth declines at 35.5°C as indicated in Figure 1 (with growth calculated from OD_750_ measurements as shown in Figure A1). As growth recovered, the temperature was increased to 36°C, and the GAI‐229 and GAI‐229 2020 strains showed adaptation as indicated with increased growth rates, from ~0.75 to ~1.3 divisions per day for GAI‐229 2020 and ~0.5 to ~1 divisions for GAI‐229. The temperature was then increased to 36.5°C. At this point, a third strain was introduced, GAI‐339, which was isolated from a batch of *N. inconspicua* cultivated outdoors in the summer of 2020, and hypothesized to have greater starting thermal tolerance than the original GAI‐229 strain due to successful outdoor growth campaigns during a relatively hot summer at the GAI Kauai Algae Farm.

Initially, at 36.5°C all strains showed sharp declines in growth, with the GAI‐229 triplicate flasks dying off completely around Day 50 of the experiment. The remaining strains, GAI‐339 and GAI‐229 2020, were maintained at this set point for 41 days, at which time the growth rate of GAI‐339 had increased from below 0.25 divisions per day to between 0.5 and 0.75 divisions per day, indicating adaptation. As a result, the next ratcheting step to 37°C was undertaken, and both strains GAI‐339 and GAI‐229 2020 again showed rapid declines in growth rates following this increase. After 5 days, GAI‐339 revived to a growth rate of around 0.5 divisions per day, while GAI‐229 2020 continued to decline until all triplicate flasks were no longer viable. With GAI‐339 as the only remaining strain to survive the ALE regime, the next ratcheting step was taken to 37.5°C. As with the prior increase, GAI‐339 showed an immediate decrease in growth rate, however, unlike the previous step, in which the strain recovered rapidly, the strain took over 20 days to recover, with a gradually increasing growth rate. This may indicate that the increased temperature during this final ratcheting step required greater adaptation than the previous step, therefore requiring a longer period to select for a beneficial phenotype from a diverse population of naturally occurring random mutations. Interestingly, while the GAI‐339 strain showed an increased growth rate over time at 37.5°C, the growth rate never increased beyond about 0.75 divisions per day. Instead, the growth rate stabilized near 0.5 divisions per day and maintained relatively consistent growth for over 100 days of continuous culturing. Compared to the initial low‐temperature growth rate of above 1.5 divisions per day, this may indicate that even with a successful ALE‐generated high‐temperature adaptation, there may be a growth limitation imposed by high temperature than cannot be overcome by the adaptation techniques used here.

Interestingly, the long duration of culturing during ALE resulted in the differentiation of the culture replicates into individual adaptive lineages. It is possible that throughout ALE, the surface‐associated (phycosphere) bacterial composition of each culture may have changed, which could have contributed to the observed changes in replicate growth rates. These growth rate changes became especially apparent after day 120 (approximately 22 generations of ALE), where GAI‐339 replicates 1 and 3 began to adapt to the recently increased temperature, whereas replicate 2 did not. This result is visible in the large error (representing one standard deviation) shown in the average OD_750_ calculated growth rate during that period, as seen in Figure 1 designated by an arrow. As a result, replicate 2 was discontinued on Day 133. The surviving two replicates adapted to the 37.5°C temperature that proved lethal for replicate 2, and two new replicates (labeled 339 1A and 339 3A) were generated from them on Day 157. Therefore, replicates 339 1 and 1A are considered one adaptive lineage, and replicates 339 3 and 3A are considered another. Despite their adaptations, all of the new replicates had similar growth rates of ~0.4 divisions per day following 287 days of ALE. At the final ratchet temperature of 37.5°C, it was hypothesized that a population of GAI‐339 had been isolated with greater thermal tolerance than the parent GAI‐339 [wild‐type (WT)] strain.

**Figure 2:**
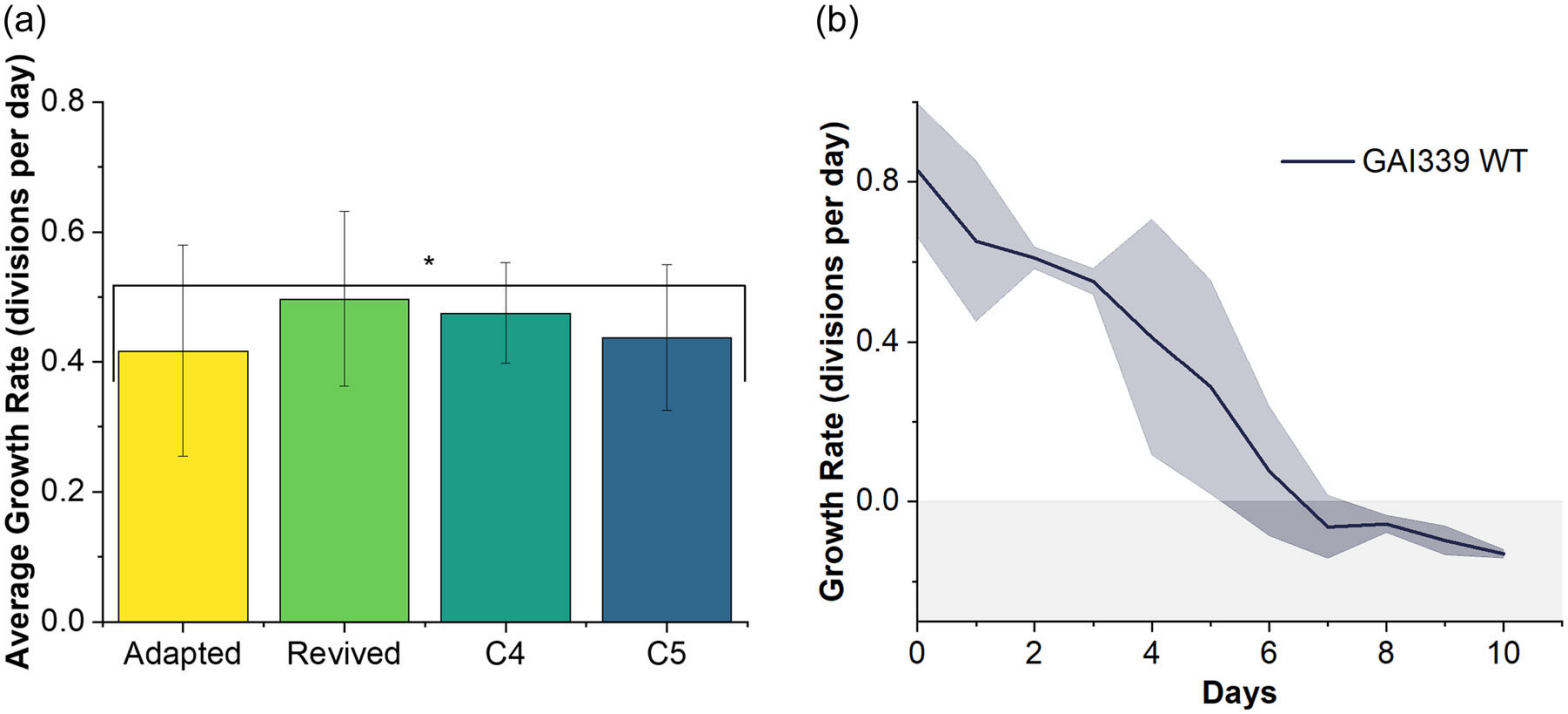
(a) Average growth rates of high temperature adapted strains of *Nitzschia inconspicua*, GAI‐339 (Adapted, biological triplicate, 137 days continuous culturing from cryo‐revival), GAI‐339 adapted and revived from cryopreservation (Revived, 21 days continuous culturing from cryo‐revival), GAI‐339 adapted clone 4 (C4, 21 days continuous culturing from cryo‐revival), GAI‐339 adapted clone 5 (C5, 17 days continuous culturing from cryo‐revival), *p > 0.05, single‐factor analysis of variance. (B) Growth rate of GAI‐339 WT (parent; unadapted) at 37.5°C with shaded blue error representing ±1 standard deviation between three biological replicates and shaded gray area highlighting values below zero.

To test this hypothesis, replicates of adapted GAI‐339 were compared to cryo‐revived GAI‐339 WT (parent) in the constant temperature incubator at 37.5°C, as shown in Figure 2. Simultaneously, the viability of the GAI‐339 WT was validated by culturing in an identical incubator set to 25°C. In the low‐temperature incubator, the GAI‐339 WT maintained growth rates between 0.5 and 1.1 divisions per day for over 20 days. In the high‐temperature incubator at 37.5°C, each of three biological replicates of GAI‐339 WT, individually revived from cryopreservation, showed immediate declines in growth, with cultures showing no viability after about 10 days, as seen in Figure 2b. This result strongly supports the hypothesis that the GAI‐339 adapted strain achieved through ALE had greater thermal tolerance than the parent GAI‐339 WT strain. To validate that the achieved adaptation was maintained through cryopreservation, a preserved aliquot of one of the triplicate cultures, GAI‐339‐1, was revived and placed in the constant temperature incubator at 37.5°C. This revived strain showed statistically similar [single factor analysis of variance (ANOVA), *p* > 0.05] growth rates to the adapted strain, around 0.5 divisions per day, which was maintained for 21 days, as shown in Figure 2a.

The thermal adaptation in *N. inconspicua* increased its upper thermal boundary by 2°C, allowing greater opportunities for siting outdoor ponds for this strain in desirable high‐temperature, high‐sunlight locations, as well as operations at shallower depths which in turn helps with light attenuation. Whereas the parent (WT) was not able to grow above 36°C, the adapted strain grew reasonably well at 37.5°C for extended campaigns (over 3 months). The achieved adaptation is maintained through cryopreservation, allowing for storage of frozen shipment of industrially relevant adapted strains. These results strongly support the hypothesis that ALE is an effective method for generating unique, thermotolerant, stable genotypes in diatomic microalgae, and highlight ALE as a means of generating algal strains that are potentially better suited for industrial cultivation.

### 3.2 Clonal isolation, morphology, and growth

**Figure 3:**
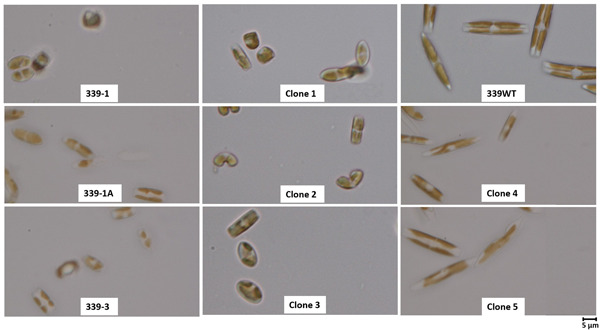
Microscopy characterization of GAI‐339 adapted clonal isolates, GAI‐339 WT, and GAI‐339 adapted lines 1, 1A, and 3 (nonclonal consortia)

ALE will inherently produce a culture with varying clonal genotypes within a mixture of cells in the evolved population, even within an adapted population with a consistent phenotype (Kassen, 2002). To isolate these potentially differing clonal genotypes, and to determine if some clonal isolates had superior performance relative to the adaptive mixture of cells, single clones were isolated from adapted GAI‐339. Once clones had been isolated and restreaked on agar plates, multiple clones were moved to liquid media for phenotypic evaluation. The following cultivation in a 25°C incubator to establish fast‐growing, dense cultures, nine clones were evaluated under high‐temperature conditions in the constant temperature incubator at 37.5°C. Three clones: 1, 2, and 9, were not tolerant to temperature and died under high‐temperature cultivation. The remaining six clones showed relatively similar growth rates of ~0.5 divisions per day. Two clones were of particular interest, clones 4 and 5, due to observed larger cell sizes, as shown in Figure 3. The average growth of clone 4, clone 5, GAI‐339 adapted, and GAI‐339 adapted cryo‐revived was compared over at least 17 days, with all strains showing statistically similar growth rates (single factor ANOVA, *p* > 0.05), as shown in Figure 2a.

Despite similar adaptive phenotypes, a microscopic evaluation showed significantly variable cellular morphologies, as shown in Figure 3. The parent population, GAI‐339 WT, contained large cells with greater than 20 µm lengths. Comparatively, the adapted lines showed smaller cells with lengths of 5–8 µm. It is well documented in the literature that diatom cell size may decrease over time as the cells undergo progressive cell division cycles (D′Alelio et al., 2009, 2010; Davidovich & Bates, 1998; Montagnes & Franklin, 2002). Many diatoms have been shown to utilize sexual reproduction as a means of restoring the cellular size, although the means by which a given diatom species initiates the sexual reproduction process is often not established (D′Alelio et al., 2009; Davidovich & Bates, 1998; Montagnes & Franklin, 2002; Rovira et al., 2015; Sarno et al., 2010). Following the clonal isolation process, during which cells were incubated on agar plates for up to 2 weeks, and once clones were transferred into liquid media, cell size was observed through microscopy (3–4 weeks following initial clonal isolation). Interestingly, clones 4 and 5, isolated from GAI‐339 adapted line 1 (GAI‐339 1), exhibited cell sizes similar to the parent GAI‐339 WT, with cell lengths of 20 µm or greater. It is hypothesized that part of the clonal isolation process may have triggered a mating event in some of the clonal populations, resulting in clones 4 and 5 with restored large cell sizes. Further investigation is needed to determine if a certain aspect of the clonal isolation process can be utilized to induce sexual reproduction in the laboratory in this species of *Nitzschia*.

### 3.3 Pond‐relevance of constant temperature‐adapted strains

Constant temperature incubator‐based ALE indicates that *N. inconspicua* is capable of adapting to thermal stress. To evaluate the utility of a constant temperature‐adapted strain, clone 4 was sent to the GAI Kauai Algae Farm in October 2021 in Lihue, Hawaii, for outdoor cultivation. The fall outdoor growth season was too cool to consistently reach pond temperatures greater than 37°C. To simulate hotter cultivation conditions, small aquarium heaters were added to two of the ponds to evaluate the impact of increased temperature on each strain. The adapted GAI‐339 clone 4 was screened side‐by‐side with the GAI‐339 WT control strain to compare the performance of each strain during the specific environmental conditions present during the test period. Preliminary results from outdoor cultivation show a 66% increase in average biomass productivity of the GAI‐339 adapted clone 4 compared to the parent GAI‐339 WT over 10 days in heated conditions, and a 21% increase in biomass productivity of the GAI‐339 adapted clone 4 compared to the parent GAI‐339 WT over 10 days in unheated pond conditions. While this preliminary pond data is promising in realizing an outdoor benefit to ALE in diatoms, we were unable to attain outdoor replicates due to logistical constraints, and these results must be repeated with additional replicates to establish statistical significance.

As documented throughout ALE literature, it is critical to design ALE experiments in a very targeted manner, because an adaptation to one condition may not translate to another condition, even if an adaptation to the former should hypothetically be beneficial to the latter (Kato et al., 2017; LaPanse et al., 2020; McDonald, 2019; Perrineau et al., 2014; Sun et al., 2016). With this in mind, future ALE experiments should preferentially be conducted using diel light and temperature to select genotypes more likely to be beneficial to pond conditions. Recent work in *Picochlorum spp.* shows that bioreactor‐based adaptation is successful in increasing the high‐temperature boundary of microalgae (Barten et al., 2022). Additionally, for diatom‐based ALE, knowledge of cell size and reproductive cycle timing will also be an important experimental design parameter to account for.

## 4 CONCLUSION

Through the use of a ratcheting ALE protocol, the temperature tolerance of *N. inconspicua* was increased from 35.5°C to 37.5°C following over 300 days of flask cultivation. This adaptation represents a systematic increase in the upper‐temperature boundary for this organism, with adapted lines able to survive at a temperature that was lethal to the parent (WT) strain. The final temperature adaptation was maintained through cryopreservation and was observed in multiple clonal isolates, including multiple clonal isolates with significantly increased cell size, indicating the potential occurrence of a sexual cycle during the clonal isolation process. Preliminary outdoor data indicated that one of the adapted clones, clone 4, may show an outdoor productivity benefit under high‐temperature conditions when compared to the parent (WT) strain; however, replicate testing is needed to verify these observations. Although outdoor testing was undertaken during cooler temperatures at the Kauai Algae Farm, the high‐temperature adaptation achieved may prove especially beneficial in allowing for the cultivation of this strain at algal cultivation sites with warm climates and consistent sunlight. All of these data suggest that *N. inconspicua* readily adapts to thermal pressures using ALE, and cultivation under outdoor‐simulating conditions within the laboratory, including diel light and temperature, will be beneficial going forward to optimize the pond‐relevance of generated adaptive phenotypes.

## AUTHOR CONTRIBUTIONS

**Alaina J. LaPanse**: Conceptualization (equal); Data curation (lead); Formal analysis (lead); Methodology (lead); Validation (equal); Writing – original draft (lead); Writing – review & editing (equal). **Tyson A. Burch**: Data curation (equal); Formal analysis (equal); Methodology (equal); Validation (equal); Writing – review & editing (equal). **Jacob M. Tamburro**: Data curation (equal); Investigation (equal); Methodology (equal); Writing – review & editing (equal). **Jesse C. Traller**: Conceptualization (equal); Funding acquisition (equal); Project administration (equal); Resources (equal); Supervision (equal); Writing – review & editing (equal). **Agnieszka Pinowska**: Funding acquisition (equal); Project administration (equal); Resources (equal); Supervision (equal); Validation (equal); Writing – review & editing (equal). **Matthew C. Posewitz**: Conceptualization (equal); Formal analysis (equal); Funding acquisition (lead); Methodology (equal); Project administration (lead); Resources (lead); Supervision (lead); Validation (equal); Writing – original draft (equal).

## CONFLICT OF INTEREST

Research at the Colorado School of Mines was supported by a research grant from the United States Department of Energy.

## ETHICS STATEMENT

None required.

## Data Availability

All data are provided in the results and appendix data sections of this paper.
